# Association between vitamin D deficiency and mortality in critically ill adult patients: a meta-analysis of cohort studies

**DOI:** 10.1186/s13054-014-0684-9

**Published:** 2014-12-12

**Authors:** Yan-Peng Zhang, You-Dong Wan, Tong-Wen Sun, Quan-Cheng Kan, Le-Xin Wang

**Affiliations:** Department of Integrated Intensive Care Unit, the First Affiliated Hospital, Zhengzhou University, 1 Jianshe East Road, Zhengzhou, 450052 China; Pharmaceutical Department, the First Affiliated Hospital, Zhengzhou University, 1 Jianshe East Road, Zhengzhou, 450052 China; School of Biomedical Science, Charles Sturt University, Wagga Wagga, NSW 2650 Australia

## Abstract

**Introduction:**

Vitamin D deficiency is common in critically ill patients, and was reported to be associated with adverse outcomes. However, the effect of vitamin D deficiency on mortality in critically ill patients remains unclear.

**Methods:**

We searched PubMed and EMBASE from the inception to July 2014 for cohort studies to assess the effect of vitamin D deficiency on the incidence of mortality in critically ill patients. Mortality-specific odds ratio (OR) with 95% confidence interval (CI) were pooled with a random- or fixed-effect models when appropriate.

**Results:**

Seven cohort studies with a total of 4,204 participants including 1,679 cases of vitamin D deficiency were included in this meta-analysis. Vitamin D deficiency was significantly associated with an increased hospital mortality (OR 1.76; 95% CI, 1.38 to 2.24; *P* <0.001), with very low heterogeneity (*I*^2^ = 2.3%; *P* = 0.402). The finding of increased hospital mortality in critically ill adult patients was consistently found in every stratum of our subgroup analyses.

**Conclusions:**

This meta-analysis suggests that vitamin D deficiency is associated with increased incidence of hospital mortality in critically ill adult patients.

**Electronic supplementary material:**

The online version of this article (doi:10.1186/s13054-014-0684-9) contains supplementary material, which is available to authorized users.

## Introduction

Vitamin D is a group of fat-soluble vitamins that play a significant role in the regulation of bone metabolism [1], it also plays a major role in extra-skeletal metabolic processes, such as glucose metabolism, and in many aspects of cellular functions [2,3]. Vitamin D deficiency is associated with various disorders such as diabetes, infections, myocardial infarction, autoimmune disease, chronic obstructive pulmonary disease, tuberculosis, and excess mortality in the general population [4]. The incidence of vitamin D deficiency has been reported to range from 26% to 82% in critically ill patients [5,6], and mounting evidence indicates that vitamin D deficiency is associated with adverse outcomes such as increased infection rates, prolonged length of ICU stay [7], higher in-hospital mortality [8], and increased health care costs [9]. However, Ralph *et al*. [10] did not observe an association between low levels of vitamin D and increased risk of mortality in critically ill patients, but they did find that patients with above physiological vitamin D levels had higher mortality and illness severity scores.

At present, the effect of vitamin D deficiency on outcomes such as mortality and morbidity in critically ill patients is not clear. We conducted a meta-analysis of seven cohort studies to investigate the effect of vitamin D deficiency on clinical outcomes such as hospital mortality, ICU mortality and length of ICU stay in critically ill adult patients.

## Materials and methods

### Search strategy

The search was carried out in keeping with the statement of meta-analysis of observational studies in epidemiology (MOOSE) [11]. Without language restrictions, the literature from the inception to July 2014 was searched in PubMed and EMBASE. The following search terms were used: ‘Vitamin Dʼ, ‘cholecalciferolʼ, ‘hydroxycholecalciferolsʼ, ‘ergocalciferolʼ, ‘25-hydroxyvitamin D_2_ʼ, ‘dihydrotachysterolʼ, ‘critical illʼ and ‘critically illʼ. Furthermore, we reviewed the reference lists in the retrieved articles and recent reviews to identify other potential relevant studies.

### Study selection

A published study was included if it: 1) was a cohort design; 2) explored the effect of vitamin D deficiency on mortality in the critically ill adult patients; 3) reported the adjusted effect size and its 95% CI. In the case of duplicate publication, we only included studies that were the most informative and complete.

### Data extraction and quality assessment

The following information was abstracted from all the included studies by using a standardized data collection form: study name (together with the first author’s name and publication year), study design, country, population characteristics, number of patients, number of vitamin D deficiency cases, gender composition in patients, mean age of the patients, severity of illness by simplified acute physiology score (SAPS) or acute physiology and chronic health evaluation (APACHE) score, definition of vitamin D deficiency, major clinical outcomes, and quantity score. We also checked the supplementary files, and contacted the authors for more detailed information where necessary.

We assessed the authenticity and quality of the included studies by Newcastle-Ottawa scales (NOS) [12], in which a study was judged on three broad perspectives consisting of eight items. We assigned risk-of-bias categories in accordance with the number of adequate items of NOS that were judged in each study: assessing the following categories: low risk of bias (six to eigt adequate items), medium risk of bias (four to five adequate items), high risk of bias (fewer than four adequate items), very high risk of bias (no description of methods). Two investigators (YP Zhang and YD Wan) carried out the literature search, study selection, data extraction and quality assessment independently. Any discrepancies were resolved by consensus.

### Statistical analysis

To compute a summary odds ratio (OR), we used the adjusted OR and its 95% CI in all analyses. The hazard ratio (HR) was considered as the OR directly. The heterogeneity test was conducted using the Cochran *Q*-statistic at the *P* <0.10 level of significance. We also quantified the *I*^2^ statistic, which describes the inconsistency due to the heterogeneity across studies [13-15]. An *I*^2^ value >50% indicates significant heterogeneity. The appropriate pooling method was decided according to the value of the *I*^2^ statistic: fixed-effects models for *I*^2^ < 50% and random-effects models for *I*^2^ ≥ 50% [13-15]. Prespecified subgroup analyses were performed according to minimum serum 25-hydroxyvitamin D (25(OH)D) level as the threshold to define vitamin D deficiency (20 ng/ml, 15 ng/ml, 12 ng/ml, 10 ng/ml) to examine the influence of this clinical factor on the overall risk estimation. We also conducted a sensitivity analysis by changing the pooling model (fixed-effects model or random-effects model) and using the one-study-out method and applying various exclusion criteria to test the robustness of the pooled estimate. Potential publication bias was assessed by the Egger linear regression test [16]. *P* <0.05 was considered to be representative of a significant statistical publication bias, except where otherwise specified. All statistical analyses were performed by using STATA, version 11.0 (Stata Corp).

## Results

### Study selection and study characteristics

**Figure 1:**
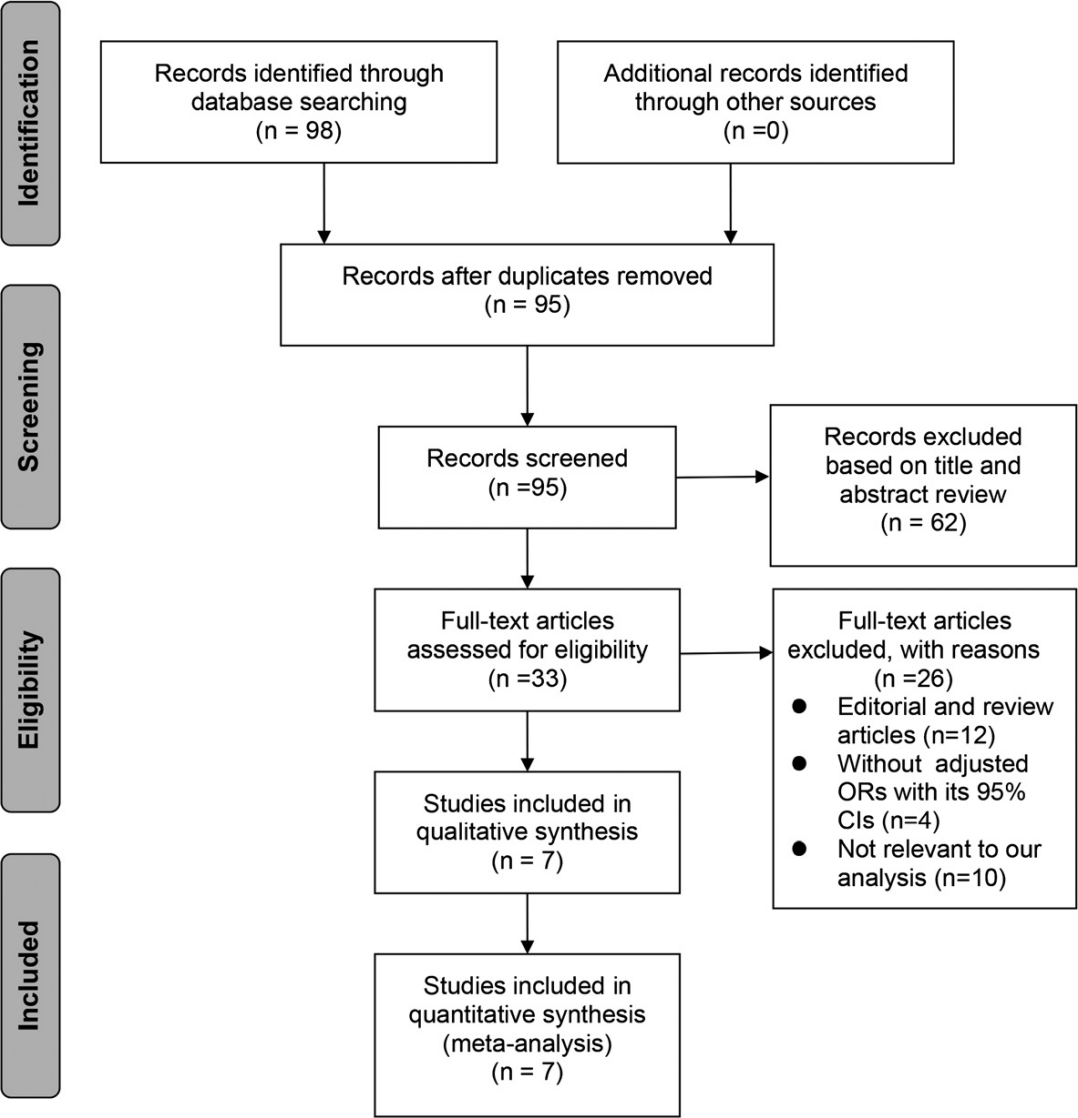
**Flow chart of article selection.** OR, odds ratio.

Our initial search yielded 98 potentially relevant publications, 3 of which were excluded for duplicate publications. We excluded 62 studies based on title and abstract review. After reviewing the full text of the remaining 33 studies, we identified 7 cohort studies in this present meta-analysis [8,17-22]. The reasons for excluding studies in the final review included: reviews and editorial articles, small sample size (n <100), not relevant to our analysis, and studies not reporting adjusted ORs of risk estimates and the 95% CI (see the detail in Figure 1).

The main characteristics of the seven studies included in this meta-analysis are shown in Table 1. In total, 4,204 participants including 1,679 cases of vitamin D deficiency were enrolled in this meta-analysis. The study-design types were as follows: single-center studies (n = 4 [8,18,19,22]), multicenter studies (n = 3 [17,20,21]), retrospective studies (n = 3 [8,17,22]), and prospective studies (n = 4 [18-21]). Six studies presented outcome of hospital mortality [8,17,18,20-22], and only two studies presented outcome of ICU mortality [19,22]. As for length of ICU stay, one study provided data expressed as mean ± SD [8], two studies did not report it [17,20], and the remaining four described the data as median [18,19,21,22]. Every single study was matched or adjusted for a wide range of potential confounders. There is no consistent definition of vitamin D deficiency between included studies. The average NOS score of the included studies was 6.6 (range from 6 to 7). An additional table file shows this in more detail (Additional file 1).

**Table 1:** **Main characteristics included in the meta-analysis**

**First author/Year of publication (reference)**	**Amrein** ***et al*** **./2014** [22]	**Aygencel** ***et al*** **./2013** [19]	**Hu** ***et al*** **./2013** [18]	**Nair** ***et al*** **./2012** [21]	**Higgins** ***et al*** **./2012** [20]	**Venkatram** ***et al*** **./2011** [8]	**Braun** ***et al*** **./2011** [17]
**Study design**	Single-center, retrospective	Single-center, prospective	Single-center, prospective	Multi-center, prospective	Multi-center, prospective	Single-center, retrospective	Multi-center, retrospective
**Country**	Austria	Turkey	China	Australia	Canada	USA	USA
**Patients**	Neurologic (30.2%), cardiac surgery (15.0%), respiratory disease (8.1%), trauma (5.8%), brain surgery (5.0%), infectious diseases, including sepsis at admission (4.4%)	Respiratory insufficiency (45%), organ dysfunctions (44%), invasive mechanical ventilation (44%), sepsis/septic shock (35.8%), renal replacement therapy (34%), nosocomial infection (35%)	Severe pneumonia with respiratory failure (47.2%), acute exacerbations of chronic obstructive pulmonary disease (19%), intra-abdominal infection (15.3%), mechanical ventilation (85.6%)	Cardiac (30%), infection/sepsis (26%), heart/lung/bone marrow transplant (21%), trauma (9%), metabolic (7%), neurological (7%)	Cardiovascular (14%), respiratory (29%), neurological (7%), metabolic (7%), gastrointestinal (3%), sepsis (5%), postoperative (35%)	Cardiac (4.8%), gastrointestinal (10.7%), metabolic (11.4%), neurological (12.5%), obstructive airway disease (13.7%), pulmonary (18.3%), others (10.9%), renal (5%), sepsis/septic shock (12.3%)	Medical (58.3%) Surgical (41.7%)
**Patients, n**	655	201	216	100	196	437	2,399
**Case patients, n (%)**	394 (60.2%)	139 (69.2%)	95 (44.0%)	24 (24.0%)	50 (25.5%)	340 (77.8%)	637 (26.6%)
**Male patients, n (%)**	412 (62.9%)	113 (56.0%)	120 (55.6%)	65 (65.0%)	121 (62.0%)	208 (47.5%)	1,030 (43.0%)
**Age, y, mean**	65 (22)^a^	66 (21)^a^	64 (25)^a^	52 ± 17^b^	64 ± 14^b^	56.6 ± 17.1^b^	64.9 ± 16.6^b^
**Severity of illness**	26 (16)^a^ (SAPS II)	23(11)^a^ (APACHE II)	21(8)^a^ (APACHE II)	21 ± 8^b^ (APACHE II)	20.2 ± 7.8^b^ (APACHE II)	67.0 ± 27.5^b^ (APACHE IV)	/
**Definition of Vitamin D deficiency, insufficiency and sufficiency**	Deficient (<20 ng/ml), insufficient (≥20 and <30 ng/ml), normal (≥30 ng/ml)	Insufficient (<20 ng/ml), sufficient (≥20 ng/ml)	Deficient (<20 ng/ml), insufficient (≥20 and <30 ng/ml), sufficient (≥30 ng/ml)	Deficient (<10 ng/ml), insufficient(10 to 20 ng/ml), sufficient(>20 ng/ml)	Deficient (≤12 ng/ml), insufficient (>12 to ≤24 ng/ml), sufficient (>24 ng/ml)	deficient (<20 ng/ml), insufficient (≥20 and <30 ng/ml), sufficient (≥30 ng/ml)	deficient(≤15 ng/mL, insufficient (16 to 29 ng/mL, sufficient (≥30 ng/ mL)
**Major clinical outcomes**	ICU and hospital mortality, ICU and hospital LOS	ICU mortality	Hospital mortality	Hospital mortality, ICU and hospital LOS	Hospital mortality, time-to-alive ICU discharge	Hospital mortality, hospital LOS	30-day, 90-day, 365-day, and in-hospital morality
**Quality score** ^**c**^	7	6	6	7	7	6	7

^a^Median (interquartile range); ^b^mean ± SD; ^c^Newcastle-Ottawa scales (NOS). SAPS, simplified acute physiology score; APACHE, acute physiology and chronic health evaluation; LOS, length of stay.

### Overall results

**Figure 2:**
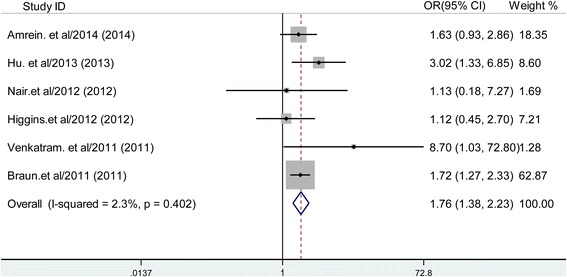
**Forest plot showing the effect of vitamin D deficiency on hospital mortality.** OR, odds ratio.

**Figure 3:**
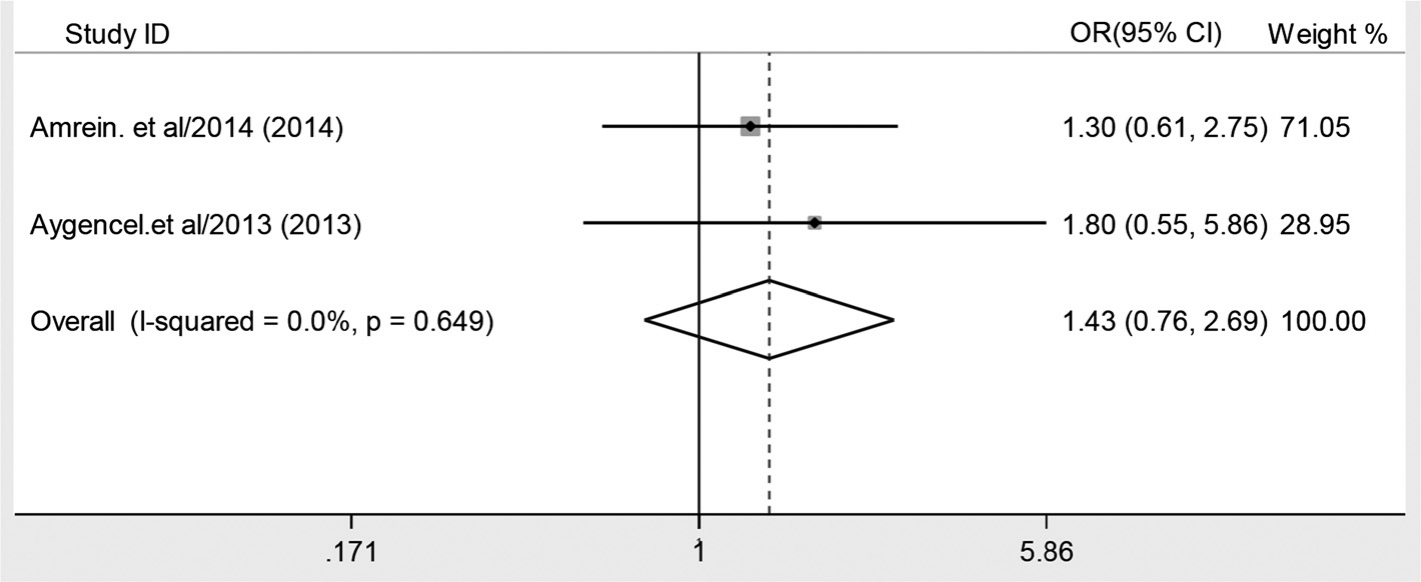
**Forest plot showing the effect of vitamin D deficiency on ICU mortality.** OR, odds ratio.

Combined by the fixed-effects model, the multivariable-adjusted OR for hospital mortality and ICU mortality for each study is shown in Figures 2 and 3, respectively. Results from six studies showed that vitamin D deficiency is associated with an increased risk of hospital mortality (OR 1.76; 95% CI, 1.38, 2.24; *P* <0. 001), with very low heterogeneity among the studies (*I*^2^ = 2.3%; *P* = 0.402). For ICU mortality, there was not enough evidence to conclude that vitamin D deficiency was not associated with increased incidence of ICU mortality in critically ill adult patients (OR 1.43; 95% CI, 0.76, 2.70; *P* = 0.271). The data for length of ICU stay for every individual study are presented in Table 2.

**Table 2:** **ICU length of stay among studies**

**Study**	**Year**	**Vitamin D classification**	**ICU length of stay (days)**
**Amrein** ***et al*** **.** [22]	2014	Deficiency, <20 ng/ml	6.9 (9.8)^a^
		insufficiency, 20 to 30 ng/ml	4.9 (7.7)^a^
		sufficiency, >30 ng/ml	5.2 (6.5)^a^
**Aygencel** ***et al*** **.** [19]	2013	Insufficiency, <20 ng/ml	9 (5, 14)^b^
		sufficiency, ≥20 ng/ml	8 (5, 13)^b^
**Hu** ***et al*** **.** [18]	2013	Deficiency, <20 ng/ml	10.5 (6.8, 25.3)^b^
		insufficiency, 20 to 30 ng/ml	8.6 (6.9, 20.5)^b^
		sufficiency, >30 ng/ml	15.2 (10.3, 23.6)^b^
**Nair** ***et al*** **.** [21]	2012	Deficiency, <10 ng/ml	12 (2, 14)^b^
		insufficiency,10 to 20 ng/ml	7 (4, 15)^b^
		sufficiency, >20 ng/ml	4 (3, 10)^b^
**Higgins** ***et al*** **.** [20]	2012	Deficient, ≤12 ng/ml	10.6 ± 8.4^c^
		insufficient, 12 to 24 ng/ml	6.8 ± 6.0^c^
		sufficient, >24 ng/ml	5.9 ± 5.4^c^
**Venkatram** ***et al*** **.** [8]	2011	Deficiency, <20 ng/ml	4.3 ± 4.5^c^
		insufficiency, 20 to 30 ng/ml	3.7 ± 3.9^c^
		sufficiency, >30 ng/ml	4.2 ± 3.7^c^
**Braun** ***et al*** **.** [17]	2011	-	-

^a^Median (interquartile range); ^b^median (Q1, Q3); ^c^mean ± SD.

### Subgroup and sensitivity analyses

Table 3 shows the results of subgroup and sensitivity analyses. Definition of vitamin D deficiency varies between studies, which may impact the overall estimates. We therefore stratified mortality rates across different studies based on vitamin D thresholds as a continuum, and the finding of increased incidence of hospital mortality in critically ill adult patients was consistently found in each stratum. Our sensitivity analyses suggested that the overall estimates were not materially altered by changing pooling models (fixed-effects model: OR 1.76; 95% CI, 1.38, 2.24 and random-effects model: OR 1.76; 95% CI, 1.37, 2.26), and also did not materially alter when any single study was removed in turn, with a range from 1.67 (95% CI, 1.30, 2.15) to 1.82 (95% CI, 1.42, 2.34).

**Table 3:** **Subgroup and sensitivity analyses for hospital mortality**

**Analyses**	**Studies, n**	**Odds ratio (95% CI)**	***Ρ***_**heterogeneity**_	***I*** ^**2**^
**Subgroup analysis**				
Total [8,17-22]	7	1.76 (1.38, 2.24)	0.402	2.3%
25(OH)D level as a cutoff for vitamin D deficiency				
20 ng/ml [8,18,22]	3	2.12 (1.35, 3.34)	0.198	38.3%
15 ng/ml [17]	1	1.72 (1.27, 2.33)	-	-
12 ng/ml [20]	1	1.12 (0.45, 2.70)	-	-
10 ng/ml [21]	1	1.13 (0.18, 7.27)	-	-
**Sensitivity analysis**				
Multicenter [17,20,21]	3	1.63 (1.23, 2.17)	0.623	0.0%
Prospective cohort [18,20,21]	3	1.83 (1.03, 3.24)	0.241	29.7%
One-study-out method				
Amrein [22]	1	1.79 (1.23, 2.70)	0.284	20.5%
Hu [18]	1	1.67 (1.30, 2.15)	0.511	0.0%
Nair [21]	1	1.77 (1.39, 2.26)	0.298	18.3%
Higgins [20]	1	1.82 (1.42, 2.34)	0.396	1.8%
Venkatram [8]	1	1.72 (1.35, 2.19)	0.571	0.0%
Braun [17]	1	1.82 (1.23, 2.70)	0.281	21.0%
Fixed-effects versus random-effects model method				
Fixed-effects model	6	1.76 (1.38, 2.24)	0.402	2.3%
Random-effects model	6	1.76 (1.37, 2.26)	0.402	2.3%

25(OH)D, 25-hydroxyvitamin D.

### Publication bias

Publication bias was assessed, but the low power with only seven studies limited the interpretability of the finding.

## Discussion

### Main findings

To the best of our knowledge, this is the first meta-analysis to explore the effect of vitamin D deficiency on mortality in critically ill adult patients. This study suggests that vitamin D deficiency is associated with increased hospital mortality in critically ill adult patients. The effect of vitamin D deficiency was observed in every stratum of our subgroup analyses. In addition, a relatively small number of samples (only two studies [19,22]) provided data on ICU mortality, and the final estimate showed there was not enough evidence to conclude that vitamin D deficiency was not associated with increased incidence of ICU mortality in critically ill adult patients (OR 1.43; 95% CI, 0.76, 2.70; *P* = 0.271).

### Possible mechanism

Although vitamin D deficiency may increase the incidence of hospital mortality in the critically ill adult patients, the reason remains unclear and can be explained by several different mechanisms. First, a range of biological responses involving immune system functions and processes in cellular growth, proliferation, and apoptosis may be influenced by vitamin D [1,23]. Vitamin D regulates the expression of the antimicrobial peptides cathelicidin (LL-37) and β-defensin, both of which have functional effectors within the immune system [24,25]. Cathelicidin can fight against a broad spectrum of infectious agents, including Gram-negative and -positive bacteria, as well as fungi [24]. Second, as vitamin D is a known link between toll-like receptors (TLR) activation and innate immunity [26], and vitamin D receptors are expressed in T cells [27], activated B cells [28], and dendritic cells [29], vitamin D deficiency may increase the risk of inflammation and sepsis in the critically ill by the suppression of immune reactivity and stimulatory effects on innate immunity [30-32]. Third, vitamin D has also been found to downregulate proinflammatory cytokines such as interferon-γ, tumor necrosis factor-α, and IL-1, IL-2, IL-6, IL-8, IL-12, as well as T helper 1 cells and B cells in the adaptive immune system [33,34]. At the same time, vitamin D upregulates anti-inflammatory cytokines, such as IL-4, IL-5 and IL-10. It promotes the expression of T-regulatory cells, which turn off the adaptive immune response [35]. These studies suggest that vitamin D deficiency disturb the innate immunity system and compromises the ability of critically ill patients to downregulate the adaptive immune response. In addition, the tissue requires more vitamin D in critically ill patients, and vitamin D deficiency may result in widespread tissue dysfunction [36]. These effects may explain why increasing mortality results from systemic inflammatory response syndrome, organ failure and metabolic dysfunction in critically ill patients.

### Clinical implications

Our findings may have several implications. As vitamin D deficiency impacts on mortality in critically ill patients, vitamin D levels may be used to predict the outcomes in critically ill patients. Unlike markers such as C-reactive protein (CRP), procalcitonin, D-dimer and leucocyte counts, which have been shown to predict disease severity in critically ill patients [37], vitamin D levels may not only predict disease severity and outcomes but also contribute to the comorbidities commonly seen in critically illness. Our meta-analysis may offer possibilities for therapeutic exploitation of vitamin D supplementation in critical illness. A recent randomized clinical trial by Amrein and colleagues [38] suggested that administration of high-dose vitamin D_3_ compared with placebo did not improve hospital length of stay and hospital mortality for vitamin D-deficient (≤20 ng/mL) patients who are critically ill. However, lower hospital mortality was observed in a subgroup of patients with severe vitamin D deficiency (≤12 ng/mL) [38]. These findings should be interpreted with caution and researchers should probe further into the effect of vitamin D supplementation on adverse outcomes, as well as the assessment of its dosing efficacy and safety.

### Strengths and limitations

The generalizability of our meta-analysis has been enhanced by its exhaustive search without language restrictions and by using the MOOSE guidelines as our systematic review methods. Also, we are able to enhance the precision of the risk estimates with our strict inclusive criteria. We included seven cohort studies with high quality, which minimized the likelihood of recall interviewer and selection biases that can be a concern in epidemiological studies. We did not find any significant heterogeneity between the studies included in our meta-analysis.

Several limitations should be acknowledged. First, some studies in this analysis did not utilize liquid chromatography-tandem mass spectrometry (LC-MS/MS) to determine 25(OH) D levels [39], which may potentially impact on the overall results. Second, vitamin D levels have seasonal variations, which have the highest mean values in August and the lowest mean levels in March [22]. Only the study by Amrein and colleagues [22] took seasonal influence into account, so we could not adjust this factor. Third, most studies were conducted in some specific ICUs, such as surgical ICU or medical ICU, which may not represent all ICU patients. Fourth, the definition of vitamin D deficiency in critically ill patients remains inconsistent, and in our meta-analysis, different criteria of vitamin D definition was used among the studies.

## Conclusions

This study showed that vitamin D deficiency is associated with increased hospital mortality in critically ill adult patients. However, there is not enough evidence to conclude that vitamin D deficiency is not associated with increased ICU mortality in critically ill adult patients, which requires more studies to probe this issue further in the future.

## Key messages

- Vitamin D deficiency is associated with increased incidence of hospital mortality in critically ill adult patients
- Current evidence about vitamin D deficiency and increased ICU mortality is still not sufficient to draw a firm conclusion
- More studies should probe further into the effect on ICU mortality in critically ill adult patients

## Additional file

Additional file 1:
**Methodological quality assessment (risk of bias) of included studies by Newcastle-Ottawa scales.**

## Abbreviations

25(OH)D: 25-hydroxyvitamin D
APACHE: acute physiology and chronic health evaluation
CRP: C-reactive protein
HR: hazard ratio
IL: interleukin
LC-MS/MS: liquid chromatography-tandem mass spectrometry
LOS: length of stay
MOOSE: meta-analysis of observational studies in epidemiology
NOS: Newcastle-Ottawa scales
OR: odds ratio
SAPS: simplified acute physiology score
TLR: toll-like receptors
