# Supplementary material for: Association between vitamin D deficiency and mortality in critically ill adult patients: a meta-analysis of cohort studies
Source: Crit Care. 2014 Dec 12;18(6):684. doi: 10.1186/s13054-014-0684-9 (PMC4274763; doi:10.1186/s13054-014-0684-9)
Supplement: Additional file 1: — Methodological quality assessment (risk of bias) of included studies by Newcastle-Ottawa scales. [file 13054_2014_684_MOESM1_ESM.doc]

| **Study** | **Selection** | | | | **Comparability** | **Outcome** | | | **Total score** |
| --- | --- | --- | --- | --- | --- | --- | --- | --- | --- |
|  | Exposed Cohort | Nonexposed Cohort | Ascertainment of exposure | Outcome of interest |  | Assessment of outcome | Length of follow-up | Adequacy of follow-up |  |
| Amrein.  et al/2014 | * | * | * | * | ** | * | - | - | 7 |
| Aygencel.  et al/2013 | - | * | * | * | ** | * | - | - | 6 |
| Hu. et al/2013 | - | * | * | * | ** | * | - | - | 6 |
| Nair.et al/2012 | * | * | * | * | ** | * | - | - | 7 |
| Higgins.et al/2012 | * | * | * | * | ** | * | - | - | 7 |
| Venkatram. et al/2011 | - | * | * | * | ** | * | - | - | 6 |
| Braun.et al/2011 | * | * | * | * | ** | * | - | - | 7 |

**Additional file 1** Methodological quality assessment (risk of bias) of included studies by Newcastle-Ottawa Scales
